# Comparative Effects of Acrolein and Glyoxal on Redox Homeostasis, Antioxidant Defense and Membrane Properties in Human Peripheral Blood Mononuclear Cells: An In Vitro Study

**DOI:** 10.3390/ijms27198706

**Published:** 2026-09-29

**Authors:** Michal Kopera, Malgorzata Adamkiewicz, Joanna Bernasinska-Slomczewska, Anna Pieniazek

**Affiliations:** 1 Department of Oncobiology and Epigenetics, Faculty of Biology and Environmental Protection, University of Lodz, ul. Pomorska 141/143, 90-236 Lodz, Poland; michal.kopera@edu.uni.lodz.pl (M.K.); joanna.bernasinska@biol.uni.lodz.pl (J.B.-S.); 2 Doctoral School of Exact and Natural Sciences, University of Lodz, 90-236 Lodz, Poland

**Keywords:** acrolein, glyoxal, human peripheral blood mononuclear cells, oxidative stress, antioxidant enzymes

## Abstract

Acrolein (ACR) and glyoxal (GO) are highly reactive carbonyls originating from endogenous metabolism, environmental pollution, and thermal food processing. This study compares the toxic mechanisms and redox effects of a 24 h exposure to ACR (30, 60, 90 µM) and GO (2, 5, 10 mM) in PBMCs. We assessed cell viability, lipid membrane fluidity, reactive oxygen/nitrogen species (ROS/RNS), free protein functional groups, reduced glutathione (GSH), non-enzymatic antioxidant capacity (NEAC), antioxidant enzyme activity (CAT, GPx), and protein expression levels (CAT, SOD1). Both aldehydes reduced viability in a concentration-dependent manner (ACR was over 30-fold more toxic than GO based on viability curves). These toxins increased membrane fluidity near the surface, while GO also altered the hydrophobic core. Both triggered a profound accumulation of ROS/RNS. At specific dose thresholds (ACR ≥ 60 µM; GO ≥ 5 mM), both toxins depleted GSH and NEAC, decreased protein thiol/amino groups, and suppressed CAT/GPx catalytic activity and CAT/SOD1 protein expression. In conclusion, ACR emerged as a markedly more potent inducer of oxidative stress and antioxidant failure than GO in PBMCs, disrupting both non-enzymatic (GSH, NEAC) and enzymatic (CAT, GPx, SOD1) defenses. These results support prioritizing ACR in exposure risk assessments and developing biomarkers of carbonyl-induced immune dysfunction.

## 1. Introduction

Numerous reactive metabolites are continuously generated during normal cellular processes, including aldehydes formed as byproducts of lipid, carbohydrate and amino acid metabolism. Under physiological conditions, their levels are controlled by enzymatic and non-enzymatic detoxification systems. However, some of these compounds, such as acrolein (ACR) and glyoxal (GO) are highly reactive. Both belong to the group of reactive carbonyl species (RCS), which possess strong electrophilic properties due to one or more carbonyl groups (C=O) [1]. Beyond endogenous production, humans are extensively exposed to ACR and GO from environmental and dietary sources [2,3,4,5].

Acrolein (2-propenal), a simple α,β-unsaturated aldehyde, is primarily generated in vivo through lipid peroxidation, and the metabolism of amino acids and polyamines [3]. Additionally, oxazaphosphorine drugs (such as cyclophosphamide, ifosfamide, and trofosfamide) metabolized by cytochrome P450 enzymes can undergo biotransformation to form ACR, contributing to elevated systemic levels [6,7]. Exogenous sources of ACR are broadly classified into environmental and dietary origins. Environmental exposure primarily occurs through tobacco smoke, air pollution, and the combustion of petroleum fuels, plastics, or wood [3]. Meanwhile, dietary exposure is driven by thermal food processing, particularly the overheating or frying of lipid-rich foods [4,8].

Glyoxal (ethanedial), a common endogenous α-dicarbonyl compound, is formed mainly during lipid peroxidation, the degradation of amino acids, glycated proteins, and nucleotides, as well as the breakdown of glucose and other carbohydrates via the Maillard reaction [2,5]. Consequently, GO serves as a primary precursor for the formation of advanced glycation end-products (AGEs) [5]. Similar to ACR, external exposure to GO is driven by both environmental factors, namely tobacco smoke and atmospheric pollution and dietary sources, such as foods processed at high temperatures [2,5].

As described above, the electrophilic nature of RCS enables them to form stable adducts with biomolecules, including proteins, lipids, and DNA [1]. Although ACR and GO are not free radicals, both can promote oxidative damage by disrupting cellular redox homeostasis [1,2,4]. Consequently, cumulative environmental and dietary exposure to these reactive carbonyl species poses a significant toxicological threat to circulating immune cells, potentially impairing their redox homeostasis and internal antioxidant defenses.

ACR preferentially targets nucleophilic cysteine thiol groups in glutathione (GSH) and various proteins [4,9]. This reaction leads to a rapid depletion of intracellular GSH, impairing thiol-dependent antioxidant defenses and compromising the cell’s capacity to neutralize reactive oxygen and nitrogen species (ROS/RNS) [10,11]. Furthermore, the modification of protein thiols alters the structure and activity of key antioxidant enzymes, mitochondrial proteins, and membrane transporters [4,11]. Consequently, ACR exposure promotes mitochondrial dysfunction, loss of mitochondrial membrane potential, disrupted energy metabolism, and increased mitochondrial ROS generation. These effects further promote lipid peroxidation, protein oxidation, and oxidative DNA damage [4,10,12]. Although GO can also react with thiol groups, it preferentially modifies the amino groups of lysine and arginine residues, leading to protein glycation, cross-linking, and AGE formation [1,2,13]. These modifications can impair protein function, including enzyme activity and alter cellular signaling pathways involved in inflammation and oxidative stress [2,14]. Like ACR, GO-mediated GSH depletion and mitochondrial impairment trigger elevated ROS production and biomolecular damage [2,13]. Moreover, GO-derived AGEs may amplify ROS-dependent inflammatory signaling through activation of AGE/RAGE-dependent pathways [13,14,15]. Thus, the toxicity of both aldehydes culminates in a self-amplifying cycle of carbonyl stress, antioxidant depletion, mitochondrial dysfunction, and macromolecular damage [1,2,4].

In addition to protein modification, GO and ACR can induce DNA damage by forming irreversible adducts or cross-links in nucleic acids. Cross-links between nucleic acid strands prevent proper DNA replication and transcription, which disrupts the integrity of the DNA structure and can lead to mutagenesis [3,16]. It was found that in patients with type 2 diabetes, the level of cross-linked DNA adducts associated with GO was significantly higher than in healthy individuals and correlated with the level of glycated hemoglobin [16]. Acrolein reacts with deoxyguanosine to form the mutagenic adduct γ-OH-Acr-dGuo. Paiano et al. reported approximately 27-fold higher adduct levels in oral cells from cigarette smokers than in nonsmokers [17], whereas Cheng et al. observed an approximately nine-fold increase in e-cigarette users compared with individuals who did not use tobacco products [18]. Ultimately, due to their high reactivity, cytotoxicity, and genotoxicity, cumulative exposure to acrolein and glyoxal significantly contributes to the development and progression of numerous diseases.

Given the widespread endogenous formation of ACR and GO in the environment, it is important to understand the specific mechanisms of their action in normal human cells. This study investigates redox related changes induced by these aldehydes in peripheral blood mononuclear cells (PBMCs). The novelty of the present study lies primarily in the direct comparison of two low-molecular-weight, reactive carbonyl species, acrolein and glyoxal, in the same primary human PBMC model and under identical experimental conditions. First, the cytotoxicity of both compounds was determined, and then the level of ROS and RNS was examined for selected concentrations of aldehydes. The cells were evaluated for changes in protein functional group levels, membrane lipid fluidity, total non-enzymatic antioxidant potential, and glutathione (GSH) levels. Furthermore, this study evaluates the concentration-dependent effects of ACR and GO on the catalytic activity of specific antioxidant enzymes (CAT and GPx), as well as the protein expression levels of CAT and SOD1. While further in vivo studies are required to establish physiological relevance, the aim of this study was to highlight the toxic potential of ACR compared to GO against PBMCs. Such findings provide a foundation that could inform future exposure risk assessments and highlight cellular redox parameters as potential targets for evaluating carbonyl-induced stress.

## 2. Results

**Figure 1:**
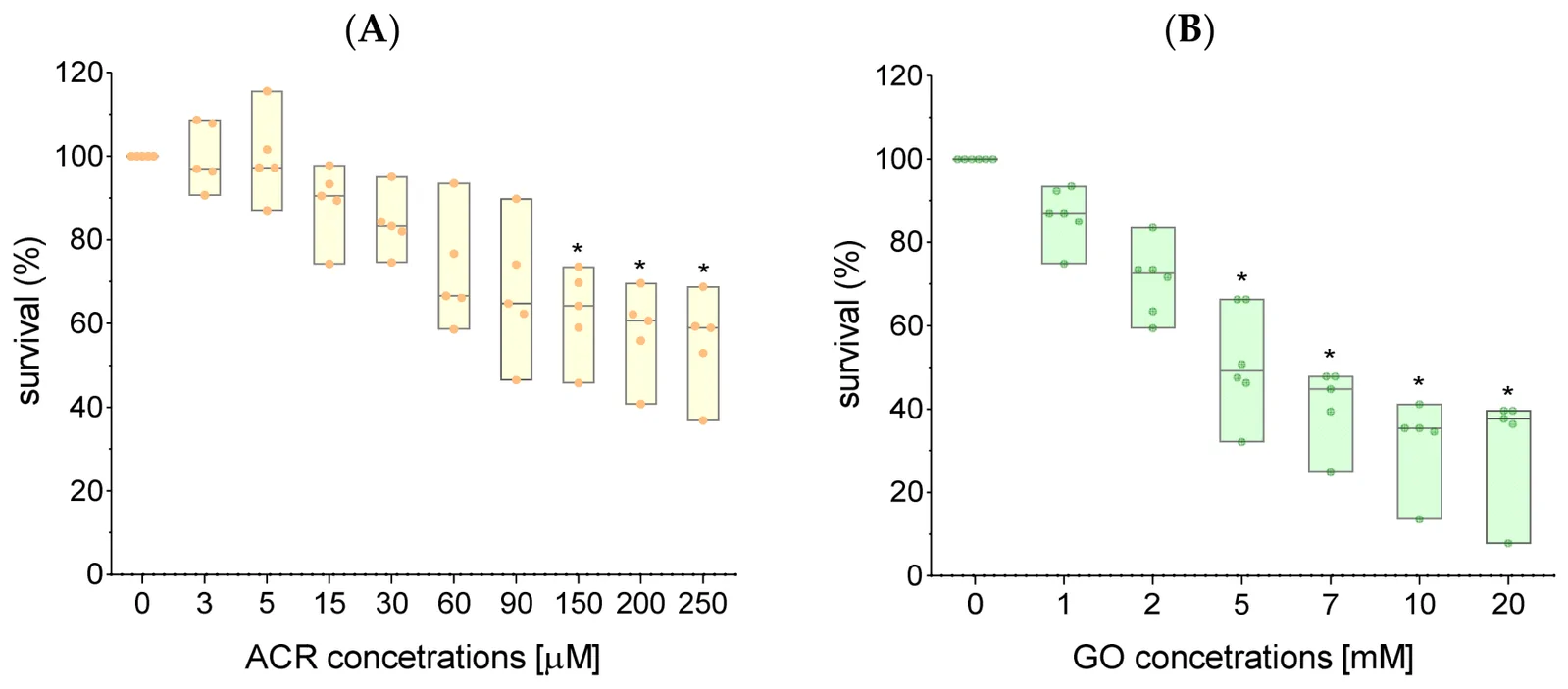
Cytotoxicity assessment of (**A**) ACR and (**B**) GO in PBMCs after 24 h of continuous incubation. Results are presented as median with a boxplot bounded minimum and maximum, and colored dots represents each individual results, n = 5. * *p* < 0.05 vs. control (Kruskal–Wallis test).

The achievement of the research objectives commenced with the assessment of cell viability in PBMCs treated with ACR and GO. The cells were exposed to the tested aldehydes for 24 h, followed by an XTT assay to evaluate their mitochondrial activity. The obtained results indicated that ACR significantly reduced cell viability at concentrations below 150 µM (Figure 1A). Conversely, GO induced a decrease in PBMCs viability at a concentration of 5 mM (Figure 1B).

**Figure 2:**
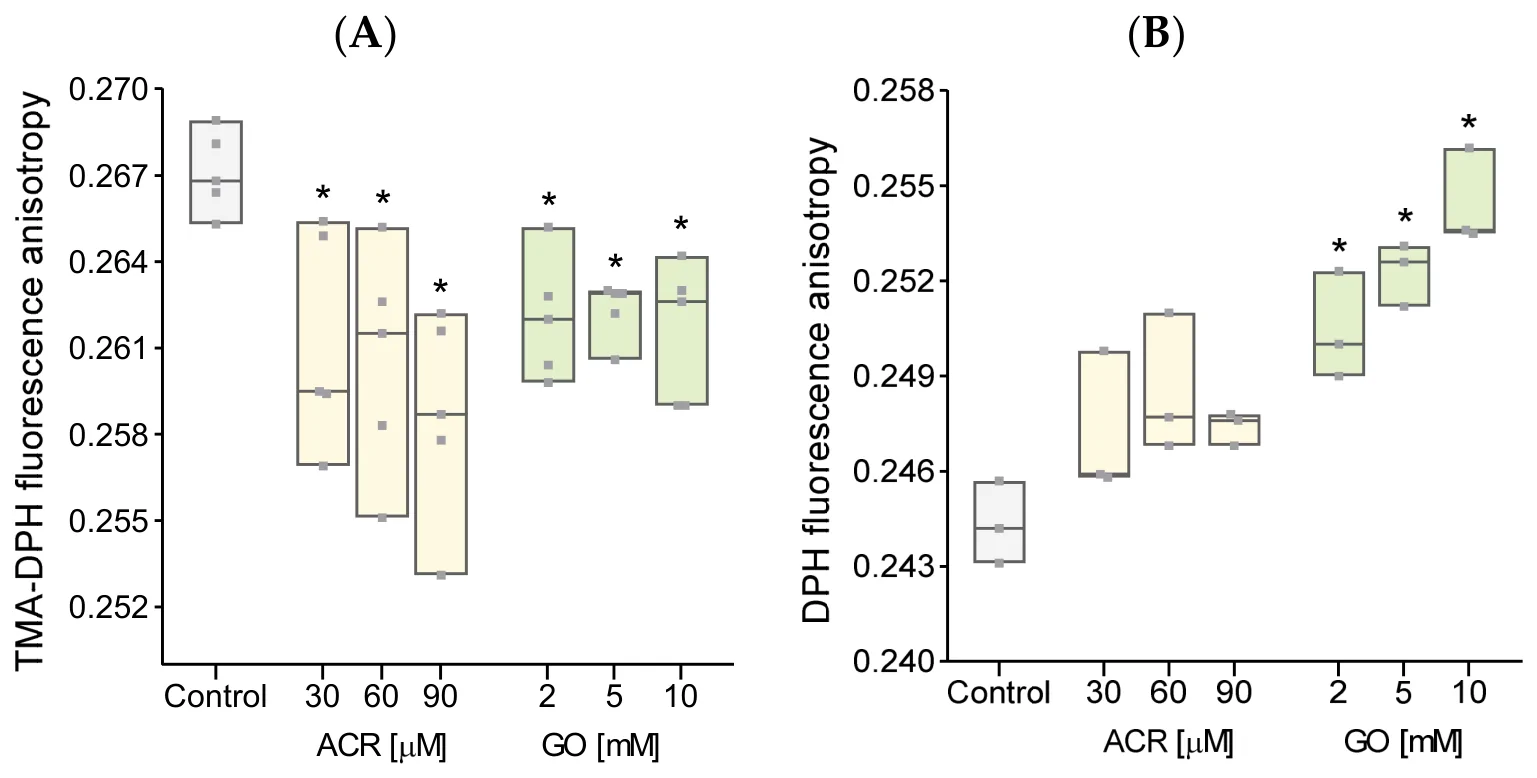
Fluorescence anisotropy of (**A**) TMA-DPH and (**B**) DPH probes incorporated into the membrane of PBMCs treated with ACR and GO. Results are presented as median with a boxplot bounded minimum and maximum, and dots represents each individual results, TMA-DPH n = 5, for DPH n = 3. * *p* < 0.05 vs. control (Tukey post hoc test).

For both studied aldehydes, transport into cells does not require specialized transport proteins; therefore, crossing the cell membrane may alter the fluidity of membrane lipids. The TMA-DPH fluorescent probe incorporates into the surface part of the outer monolayer of the cell membrane. The fluorescence anisotropy of this probe anchored in the membranes of cells treated with ACR (at all concentrations used) was significantly lower compared to the values obtained for control cells. Similarly, a decrease in fluorescence anisotropy of the TMA-DPH probe was observed in cells incubated with GO compared to control cells (Figure 2A).

The deeper regions of the outer monolayer of the cell membrane were examined using DPH. No change in the fluorescence anisotropy was observed in the membranes of ACR-treated cells. In cells treated with all tested concentrations of GO, a statistically significant increase in the fluorescence anisotropy of the membrane-incorporated DPH probe was observed compared to the control. (Figure 2B).

**Figure 3:**
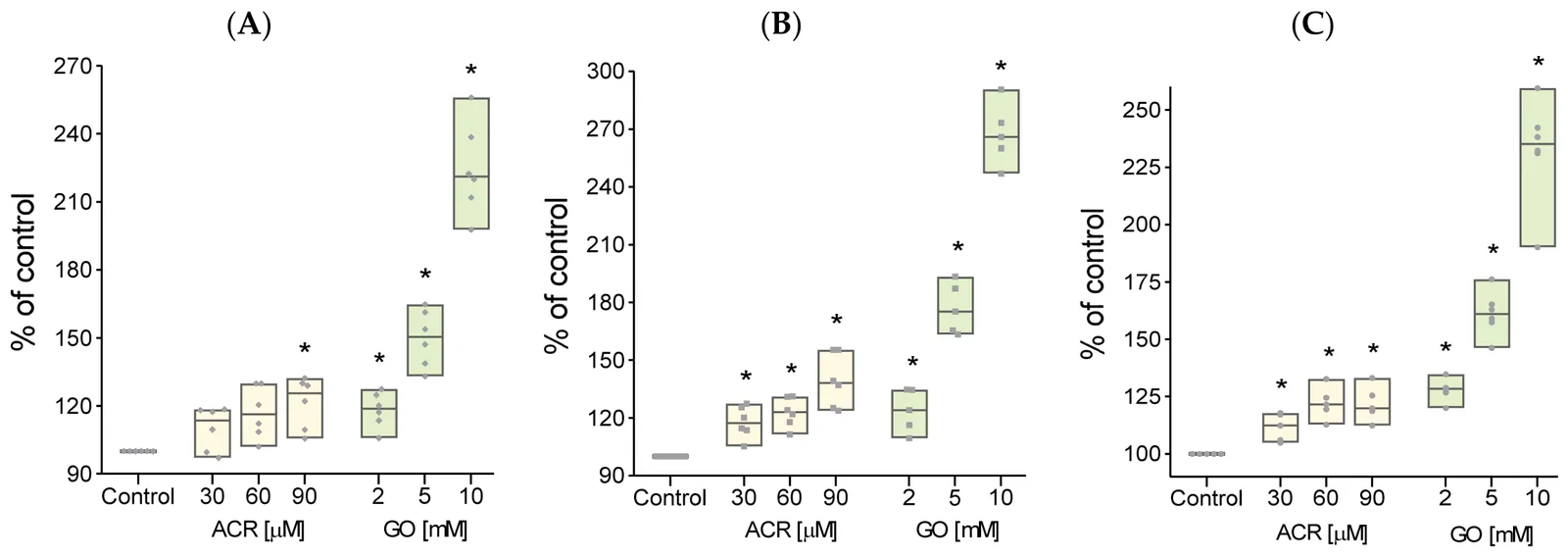
ROS and RNS levels in PBMCs treated with ACR and GO measured with fluorescent probes (**A**) H_2_DCFDA, (**B**) DHEt and (**C**) DAF-FM. Results are presented as median with a boxplot bounded minimum and maximum, and dots represents each individual results, (**A**,**B**) n = 6, for (**C**) n = 5. * *p* < 0.05 vs. control (Tukey post hoc test).

The next step in achieving the research goal was to examine the level of ROS/RNS in cells exposed to both toxins. The overall level of ROS/RNS in cells was measured using the H_2_DCFDA fluorescent probe. The obtained results indicate that both ACR and GO induce oxidative stress in cells. Statistically significant results were obtained when cells were incubated with medium and high concentrations of both toxins (Figure 3A). DHEt probe is considered one of the best and most specific fluorescent probes for detecting superoxide anion radicals. The results indicate that the level of this radical increased significantly in cells exposed to the selected toxins, even at the lowest concentration tested (Figure 3B). The fluorescent probe DAF-FM, which is specific for nitric oxide, was used to measure RNS levels. The results showed that both toxins (ACR and GO) cause a significant increase in nitric oxide levels in treated cells compared to those in the control group (Figure 3C). Statistically significant changes were observed in cells incubated with all selected toxin concentrations.

**Figure 4:**
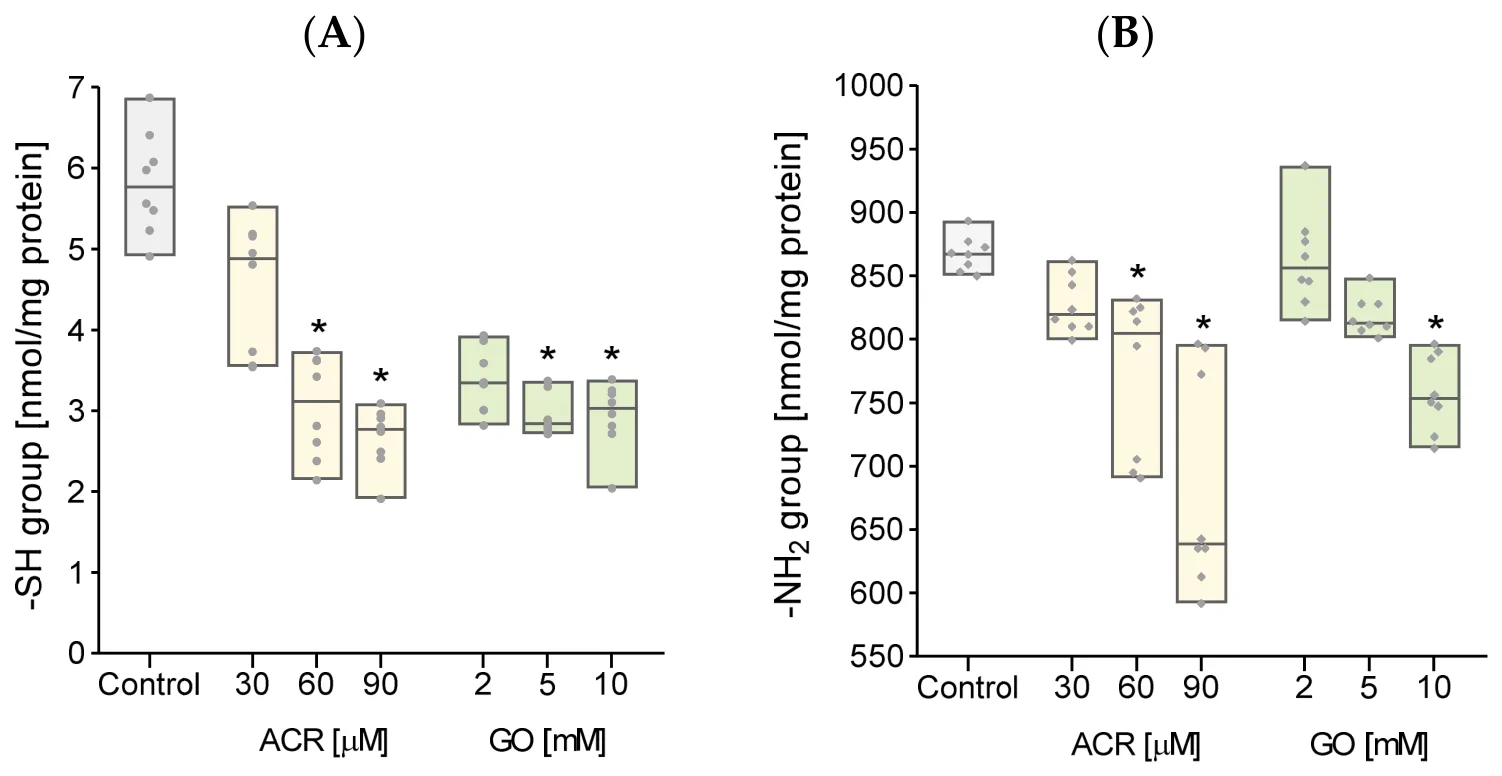
Level of free (**A**) thiol and (**B**) amino groups in PBMCs treated with ACR and GO. Results are presented as median with a boxplot bounded minimum and maximum, and dots represents each individual results, n = 8. * *p* < 0.05 vs. control (Kruskal–Wallis test).

Both ACR and GO are toxins that exhibit a high affinity for protein functional groups. Furthermore, the levels of free protein functional groups may alter in relation to the intensity of oxidative stress. Therefore, in the next stage of the study, the levels of thiol and amino groups in PBMCs proteins treated with ACR and GO were assessed. The obtained test results showed a significant decrease in the level of thiol groups in cells incubated with ACR (60 and 90 μM) and GO (5 and 10 mM) in comparison to control level (Figure 4A). The levels of amino groups were significantly reduced in cells treated with ACR at concentrations of 60 μM and 90 μM, whereas in GO-treated cells, a significant decrease in this parameter was observed only at the highest concentration (10 mM) compared to the control values (Figure 4B).

**Figure 5:**
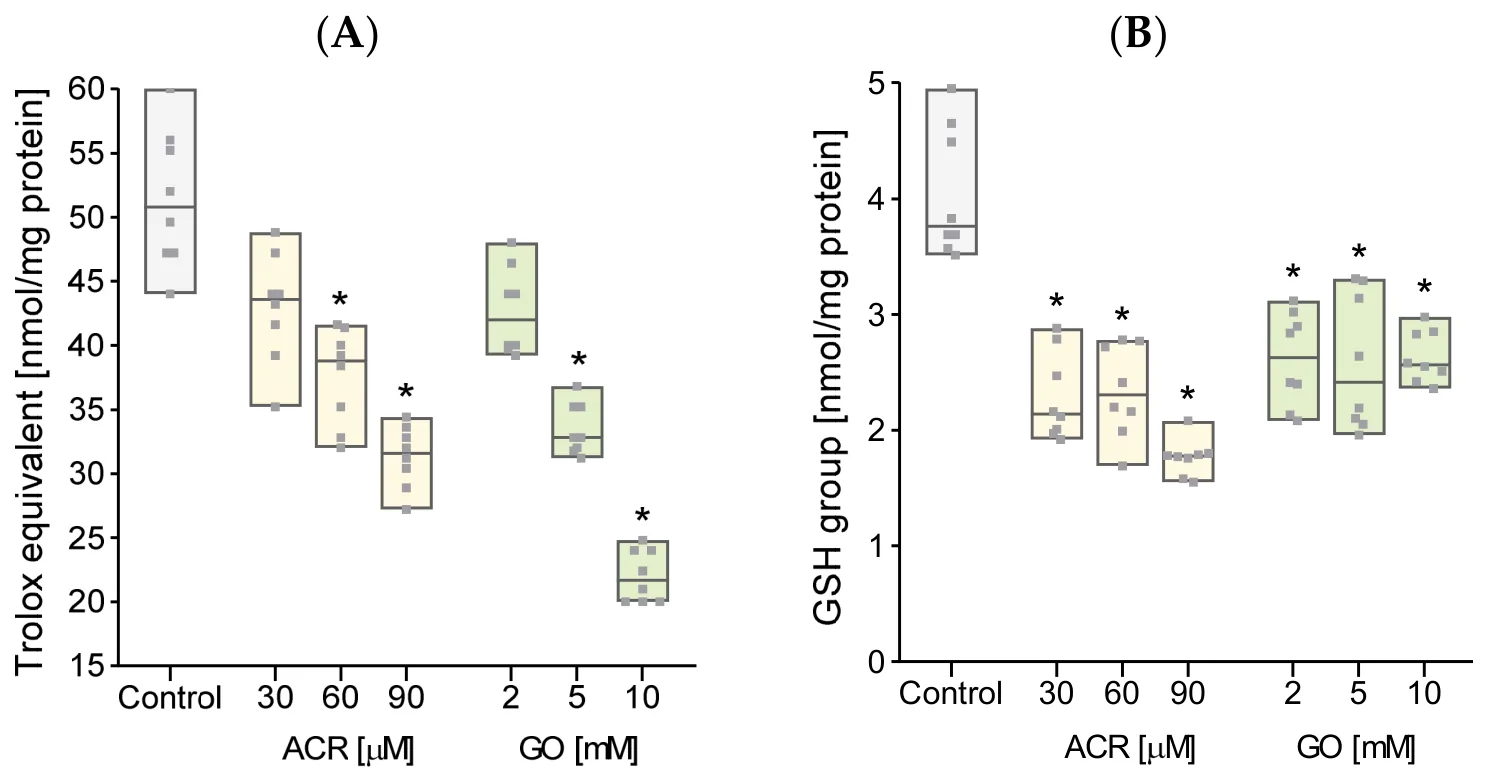
(**A**) Total non-enzymatic antioxidant capacity and (**B**) reduced glutathione levels in PBMCs treated with ACR and GO. Results are presented as median with a boxplot bounded minimum and maximum, and dots represents each individual results, n = 8. * *p* < 0.05 vs. control (Kruskal–Wallis test).

Increased cellular ROS/RNS production may alter the activity of enzymatic and non-enzymatic antioxidant defenses. Reduced glutathione levels and total non-enzymatic antioxidant capacity (NEAC) were evaluated in ACR- and GO-treated cells. The obtained research results indicate that both ACR and GO contribute to the reduction of NEAC in cells. A statistically significant decrease in this parameter compared to the control was observed after incubating PBMCs with ACR at concentrations of 60 µM and 90 µM, as well as after incubating PBMCs with GO at concentrations of 5 mM and 10 mM (Figure 5A). One of the most important endogenous antioxidants in cells is glutathione. The study of GSH levels in cells treated with ACR and GO showed that both toxins caused a significant decrease in this antioxidant compared to the control, even at the lowest concentrations tested (Figure 5B). It is worth noting that the intracellular GSH pool did not alter as the toxin concentration increased, maintaining the level reached at the lowest concentrations.

**Figure 6:**
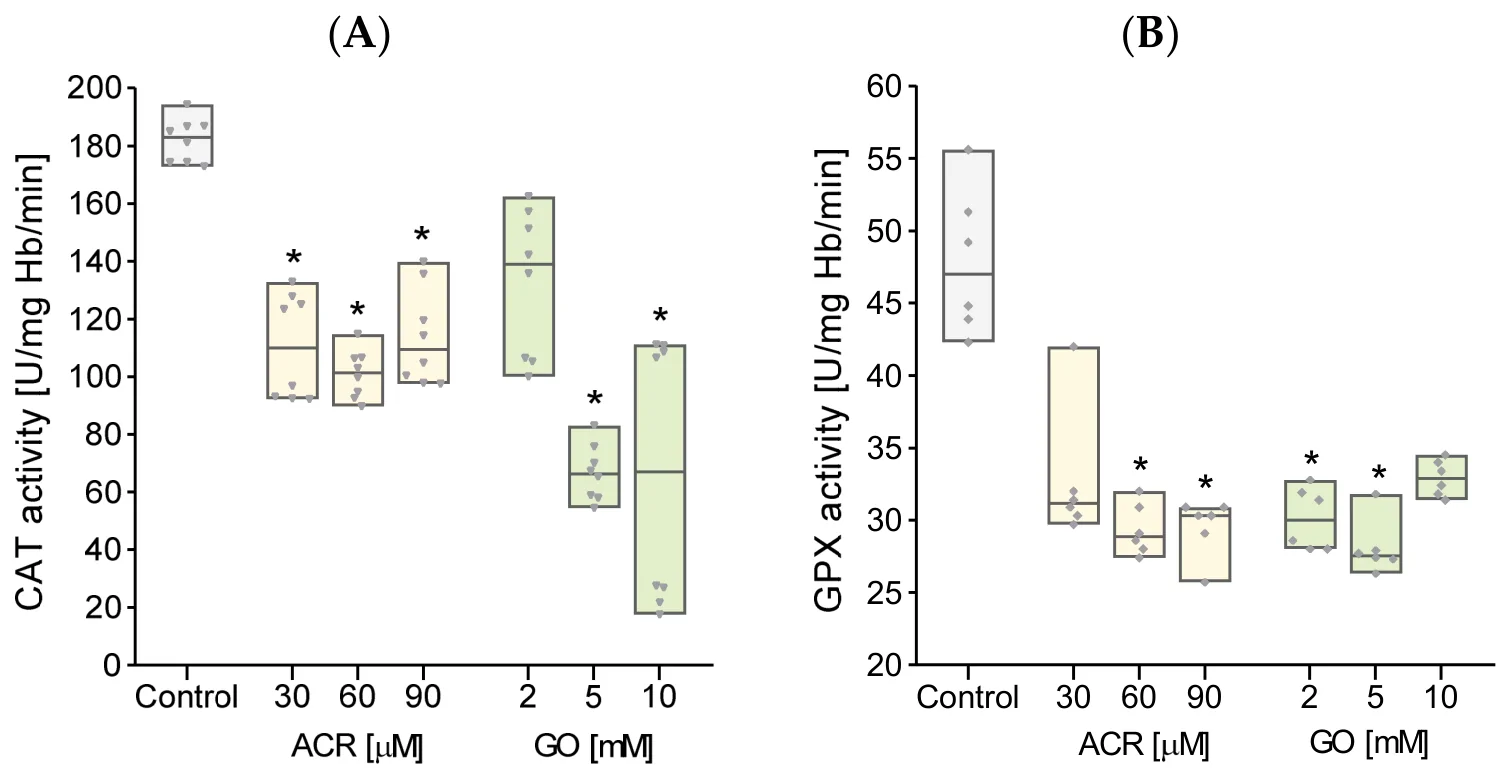
Activity of (**A**) catalase and (**B**) glutathione peroxidase in PBMCs treated with ACR and GO. Results are presented as median with a boxplot bounded minimum and maximum, and dots represents each individual results, CAT n = 8, for GPx n = 6. * *p* < 0.05 vs. control (Kruskal–Wallis test).

The antioxidant system of cells also includes a number of enzymes that inactivate ROS. In this study, the activities of catalase and glutathione peroxidase were analyzed in cells treated with ACR and GO. ACR caused a significant decrease in catalase activity in PBMCs compared to the values obtained for control cells. On the other hand, GO caused a significant decrease in the activity of this enzyme only at the two highest concentrations tested, compared to the control (Figure 6A). Similarly to catalase, both ACR and GO contributed to the reduction of glutathione peroxidase activity in PBMCs compared to the values obtained for control cells. For both toxins, a statistically significant decrease in the activity of this enzyme was observed at the two highest concentrations used (60 and 90 µM for ACR and 5 and 10 mM for GO) (Figure 6B).

**Figure 7:**
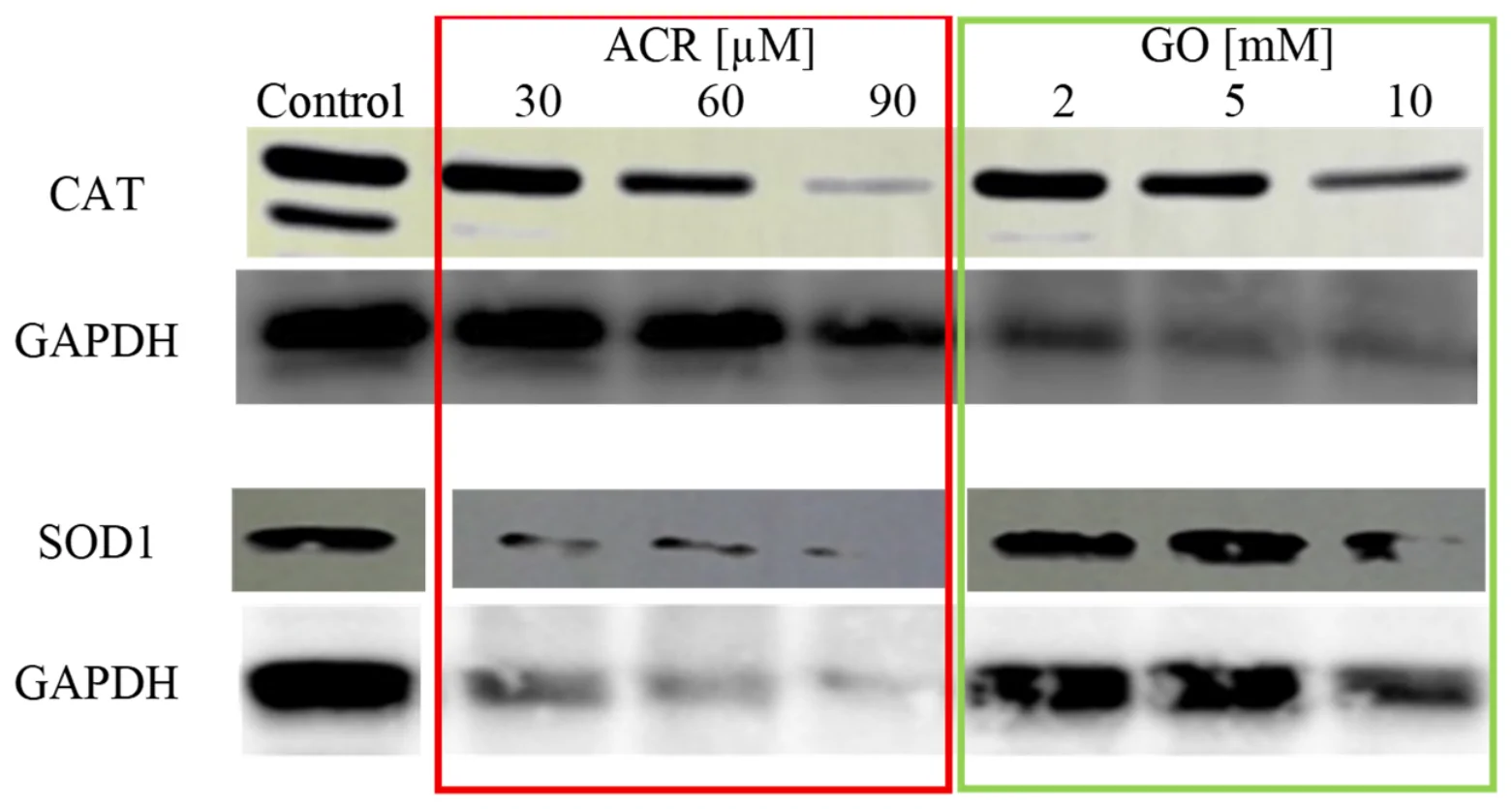
The expression of catalase, superoxide dismutase and GAPDH protein in PBMCs treated with ACR or GO for 24 h. The proteins were separated by SDS-PAGE, and their levels were analyzed by Western blot (n = 3).

An important factor in the oxidation-reduction balance of cells is the proper expression of genes encoding antioxidant enzymes. In this study, the expression of superoxide dismutase (SOD1) and catalase (CAT) in PBMCs treated with ACR and GO was examined (Figure 7). The results demonstrated that ACR significantly reduced the expression of catalase at concentrations of 60 and 90 µM, and superoxide dismutase at all analyzed concentrations. In turn, GO decreased the expression of both enzymes in PBMCs at concentrations above 5 mM. The glyceraldehyde 3-phosphate dehydrogenase (GAPDH) was used as a reference protein to normalize the expression results of the tested enzymes. We observed disturbances in the expression of this protein. To account for variations in sample preparation and protein concentration, Ponceau S staining was also performed (Online Resource).

## 3. Discussion

ACR and GO are not only compounds to which humans are exposed through inhalation or ingestion; they are also compounds that are products of cell metabolism. Excessive exposure to these aldehydes may cause and/or exacerbate the symptoms of many diseases. Therefore, understanding the mechanisms of action of both toxins may contribute to the development of ways to prevent the effects of their action in cells. PBMCs were used in the study to reflect the effects of these toxins on a healthy body. Exposure of cells for 24 h to ACR at concentrations above 150 µM caused a significant decrease in their survival. In turn, GO significantly reduces cell survival when used at concentrations above 5 mM. For A549 cells, Sakamoto et al. determined the IC50 parameter to be 25 µM after 72 h of incubation with ACR [19]. The same authors also conducted studies on cell survival at different times of exposure to the toxin. They thus confirmed that the effects of this toxin may depend on the time of exposure. Another group of scientists performed a survival test on three cell lines exposed to ACR for 24 h. They obtained the IC50 value for the Caco-2 cell line was 60 µM, and for the GES-1 line it was 54 µM, while the HUVEC line proved to be the most sensitive, with an IC50 of 16 µM [20]. Another study also examined the toxicity of GO to cells mimicking the human intestine (Caco-2). The authors of another study demonstrated a statistically significant decrease in cell viability in the presence of GO at concentrations above 0.8 mM during a 24 h exposure [21]. In turn, Liu et al. showed that GO at a concentration of 0.25 mM significantly reduced the survival of HEK293 (Human Embryonic Kidney 293) after 24 h treatment [13]. Additionally, Gurney et al. suggest that GO may loss of trans-endothelial electrical resistance), enhanced transcellular permeability of dextran, and caused cytoskeletal reorganization [22]. It is worth noting here that each type of cell has different functions and metabolism, which means that it may also show different sensitivity to toxic factors. Studies on cytotoxicity often describe the IC50 parameter, which determines the concentration of a substance that causes a 50% reduction in cell viability. Because ACR treatment (up to 250 µM) reduced viability only to ~55%, its IC50 was omitted. Conversely, GO yielded an IC50 = 6.04 ± 1.4 mM). However, simultaneous studies on ACR and GO using the same cell type, alongside the analysis of existing literature, confirm that ACR is significantly more toxic than GO to PBMCs.

ACR has been proposed to cross biological membranes by passive diffusion, although its uptake mechanism has not been directly established in PBMCs [23,24]. GO can also cross cellular membranes; studies in a human Caco-2 model indicate contributions of both passive and active transport, whereas its uptake mechanism in PBMCs remains unknown [25]. Therefore, a direct relationship between aldehyde transport and the observed membrane alterations cannot be established from the present data. Membrane organization was assessed using the TMA-DPH and DPH fluorescence probes, which preferentially report properties of different membrane regions. Decreased TMA-DPH anisotropy after both ACR and GO exposure is consistent with increased probe mobility in the membrane interfacial region. In contrast, DPH anisotropy was unchanged after ACR treatment but increased after GO exposure, indicating restricted probe mobility and greater lipid ordering in the more hydrophobic region. Thus, the observed effects should be interpreted as probe-specific, region-dependent alterations in membrane organization rather than as a uniform change in membrane fluidity across the lipid bilayer. Previous studies have demonstrated increased osmotic sensitivity in erythrocytes treated with both ACR and GO, as well as alterations in membrane fluidity induced by ACR [26,27]. One of the reasons for changes in the rheology of the cell membrane may be conformational changes in membrane proteins that interact with the tested toxins. Other factors responsible for this phenomenon may include the oxidation of lipids and membrane proteins, induced by oxidative stress. Our research has demonstrated that the presence of tested toxins in cells induces the production of ROS and RNS. Our results confirm earlier in vitro findings that ACR exposure induces an upregulation of cellular ROS production [28]. The authors of this study also observed a significant increase in Zn^2+^ in cells exposed to ACR. A sudden rise in zinc ion concentration can disrupt proper electron transport in the mitochondrial respiratory chain, resulting in increased ROS levels [28]. Similar results of the study of ROS levels in cells treated with ACR were also obtained by Liu et al. [12]. The authors of this work also observed changes in the mitochondrial potential of cells exposed to ACR. This may also explain the disruption of electron flow in the respiratory chain and the induction of oxidative stress. The increase in ROS levels in HEK293 cells treated with GO was also observed by Liu et al. [13]. The authors of this study also demonstrated an increased expression of the RAGE protein, which binds, among others, advanced glycation end-products (AGEs). Additionally, they observed an elevated Bax/Bcl-2 ratio and alterations in other protein expressions, suggesting that GO induces apoptosis via the mitochondrial pathway [13].

Elevated ROS production in cells is associated with the oxidation of cellular biomolecules. In proteins, the -SH and -NH_2_ functional groups are the most susceptible to these processes. Both ACR and GO are compounds involved in the formation of carbonyl stress in cells. They show a high affinity to these functional groups and thus contribute to the structural and functional modification of proteins [29,30]. Hence, the significant decrease in the level of thiol and amino groups in PBMCs proteins exposed to ACR and GO, obtained in this study, may confirm the high reactivity of these toxins. A decrease in the level of thiol and amine groups in erythrocytes treated with ACR and GO was also observed in our earlier studies [26,27]. Changes in the levels of thiol and amino groups are not limited to protein modifications. In cells treated with ACR and GO, the decrease in the level of GSH may result from the induction of oxidative stress and increased activity of the antioxidant system, as well as from the direct interaction of this compound with the studied toxins. ACR is mainly eliminated from the body by conjugating with GSH in the liver. The ACR-GSH complex is enzymatically metabolized via the removal of the γ-glutamic acid and glycine residues within the liver and kidneys, respectively [24]. In the case of GO, as Nomi and colleagues suggest, its primary binding site in GSH is the N-terminal amine group. This reaction ultimately results in two products: N-Glycoloyl-glutamylcysteinylglycine and N-[3-(2,5-Dioxomorpholin-3-yl)-propanoyl]cysteinylglycine [31]. The antioxidant barrier of cells includes an enzymatic and non-enzymatic system. In this study, we showed that ACR and GO contribute to the reduction of catalase and glutathione peroxidase activity in cells. The obtained results for ACR are in agreement with previous findings in erythrocytes [26] and ARPE-19 cells [32]. The reduction of catalase and glutathione peroxidase activity in Caco-2 cells treated with GO was also observed in the work of Mu et al. [21].

The effectiveness of the antioxidant system is highly dependent on the expression of genes that encode enzymes. Nrf2 is as a key transcriptional regulator of antioxidant response element (ARE)-mediated gene expression and phase II detoxifying antioxidant enzymes. However, Nrf2 activation patterns exhibit marked cell-type-specific and context-dependent variability under aldehyde exposure. For instance, Jia et al. showed that ACR exposure significantly decrease total and nuclear Nrf2 expression in ARPE-19 cells [32], whereas Mu et al. observed an increase in Nrf2 expression in Caco-2 cells treated with 1.6 mM GO for 24 h [21]. Although Nrf2 pathway status was not directly measured in PBMCs in the present study, it can be hypothesized that ACR and GO might impair Nrf2-dependent signaling in this specific cell model. Such interference could hypothetically explain the observed reduction in CAT and SOD1 expression and activity at higher toxin concentrations. Nevertheless, because aldehyde-induced Nrf2 responses vary considerably depending on the cell type, concentration, and exposure duration—ranging from Nrf2 activation under moderate hormetic stress to its degradation or pathway suppression at high concentrations—further studies evaluating Nrf2 nuclear translocation or ARE-reporter activity in PBMCs are required to confirm this proposed mechanism.

The reference protein for the analysis of antioxidant enzyme expression was GAPDH (glyceraldehyde-3-phosphate dehydrogenase). Western blot analysis showed changes in this protein in cells treated with ACR and GO. The results are in the line with studies by Kashiwagi and Igarashi showed that glyceraldehyde-3-phosphate dehydrogenase (GAPDH), actin, α- and β-tubulin proteins, or apolipoprotein B-100 in LDL are severely damaged by ACR conjugation [33]. Furthermore, Gurney et al. showed that GO also caused cytoskeletal protein reorganization in cells [22]. Its modification by reactive aldehydes may alter protein stability, electrophoretic mobility or antibody recognition, making it unsuitable as a stable loading control under these experimental conditions.

While our findings offer significant insights into the comparative toxicity of ACR and GO, several limitations remain. First, the in vitro model uses supra-physiological concentrations of both aldehydes, which do not reflect typical physiological exposures and do not capture systemic complexities such as whole-body metabolism, protein binding, aldehyde clearance, and the tissue microenvironment. Second, the experiments focused on PBMCs from healthy donors, measuring all endpoints after a single 24 h exposure. This single time point limits our ability to establish causal relationships between membrane perturbation, ROS/RNS generation, antioxidant depletion, and enzyme downregulation. On the other hand, to our knowledge, such a comprehensive side-by-side comparison of acrolein and glyoxal in primary human PBMCs has not previously been reported. Finally, there are methodological limitations associated with Western blot analysis. We noted changes in GAPDH abundance and mobility under carbonyl stress, likely related to apoptosis-induced degradation. Thus, we cannot definitively conclude “structural damage” from Western blotting alone, and GAPDH should not be used as a stable loading control in these conditions. Future studies assessing protein expression under severe carbonyl stress should employ total-protein normalization to ensure accurate interpretation.

To address these limitations, our future plans will first focus on implementing repeated, low-dose chronic exposure models over extended periods. Second, we plan to expand our investigations to include human endothelial cell models, such as HUVEC or HMEC-1, to assess the broader vascular implications of carbonyl stress. Third, given the observed instability of GAPDH and its potential link to cell death pathways, future studies will incorporate a comprehensive evaluation of apoptosis. Finally, to link the observed oxidative stress to specific molecular targets and confirm our hypotheses about antioxidant downregulation, we will directly evaluate Nrf2/ARE signaling through nuclear translocation and ARE-reporter functional assays.

## 4. Materials and Methods

### 4.1. Chemicals

The following reagents were obtained from Merck (Darmstadt, Germany): 4,4′-dithiodipyridine, 2,4,6-trinitrobenzene sulfonic acid (TNBS), 2,4-dinitrophenylhydrazine (DNPH), 2,4,6-tripyridyl-S-triazine (TPTZ), glyoxal, *o*-phthalaldehyde (OPA), N-ethylmaleimide (NEM), Histopaque^®^-1077; FBS, RPMI 1640; penicillin; 2,3-bis [2-Methoxy-4-nitro-5-sulfophenyl]-2H-tetrazolium-5-carboxyanilide inner salt (XTT), phenazine methosulfate (PMS), Sigma-Aldrich (St. Louis, MO, USA) glutathion peroxidase assay kit (Catalog Number: MAK437), 2′,7′-dichlorofluorescein diacetate (H_2_DCF-DA), dihydroethidium (DHEt), Diaminofluorescein-FM (DAF-FM), fluorescent probes (DPH, TMA-DPH), RIPA buffer and secondary antibodies conjugated with alkaline phosphatase. Rabbit monoclonal antibodies specific to catalase (D4P7B, cat. no. #12980, dilution—1:2000), SOD1 (E4G1H, cat. no. #37385, dilution—1:2000), GAPDH (cat. no. #10494-1-AP, dilution—1:20,000), and secondary anti-rabbit, HRP-linked antibody (cat. no. #7074) were purchased from Cell Signaling Technology, Inc. (Danvers, MA, USA). Chemiluminescent Substrate SuperSignal™ West Pico PLUS Chemiluminescent Substrate was purchased from Thermo Fisher Scientific (Waltham, MA, USA). RPMI 1640 bicarbonate medium, fetal bovine serum (FBS), penicillin and streptomycin were from Gibco^®^ (Grand Island, NY, USA). Unless stated otherwise, all other chemicals were sourced from POCH S.A. (Gliwice, Poland).

### 4.2. Peripheral Blood Mononuclear Cells Isolation

Peripheral blood mononuclear cells (PBMCs) were obtained from human buffy coats provided by the Blood Bank in Lodz, Poland, using density-gradient centrifugation with Histopaque^®^-1077. After isolation, the cells were washed three times with phosphate-buffered saline by centrifugation at 400× *g* for 12 min. The PBMCs were then resuspended at a density of 1.5 × 10^6^ cells/mL and cultured in RPMI 1640 medium supplemented with 10% heat-inactivated fetal bovine serum, penicillin, and streptomycin.

### 4.3. Cell Viability

Cell viability of PBMCs following exposure to ACR or GO was assessed using the standard XTT colorimetric assay. Isolated PBMCs were seeded into 96-well plates at a density of 2 × 10^5^ cells per well and cultured in growth medium containing a broad range of ACR (0–250 µM) or GO (0–20 mM) concentrations for 24 h at 37 °C in a humidified incubator with 5% CO_2_. After the exposure period, the medium containing ACR or GO was replaced with fresh medium, and the cells were cultured for an additional 24 h. The XTT assay was then performed by adding freshly prepared XTT (0.05 mL, to the final concentration of 1 mg/mL) solution supplemented with PMS (to the final concentration of 0.017 mg/mL) to each well, followed by incubation for 4 h. Absorbance was measured at 495 nm using a BioTek microplate reader. Cell viability was expressed as a percentage of the control, which was set at 100%.

### 4.4. Cell Treatment

**Figure 8:**
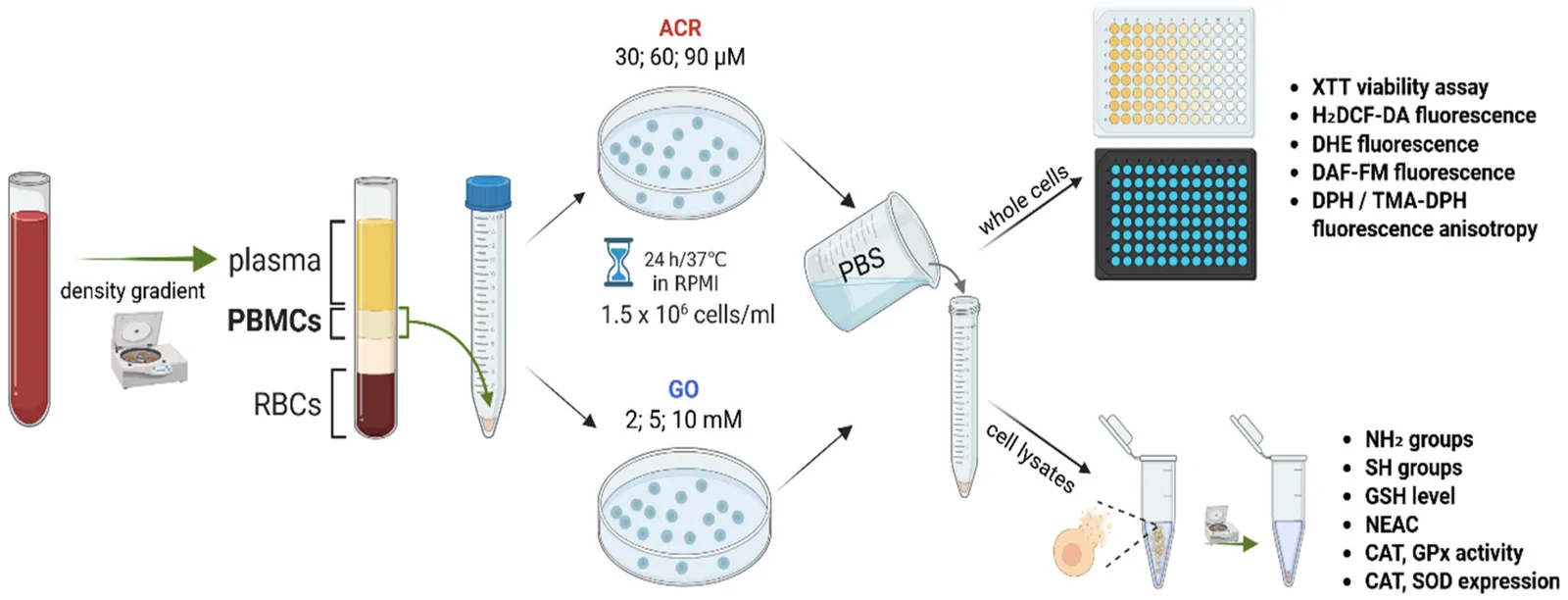
Scheme of cell preparation and experimental procedures on PBMCs subjected to ACR and GO (created by authors with BioRender.com by the authors, https://BioRender.com/nzfi4mv, accessed on 15 August 2026).

PBMCs were incubated in Petri dishes in the presence of three concentrations of GO (final concentrations: 2, 5, and 10 mM) and ACR (final concentrations: 30, 60, and 90 µM) for 24 h at 37 °C in a humidified atmosphere containing 5% CO_2_. Concentrations of ACR (30, 60, and 90 µM) and GO (2, 5, and 10 mM) were selected based on XTT cell viability assay results. For the determination of thiol groups (SH), amino groups (NH_2_) and reduced glutathione (GSH) level, antioxidant enzyme activities, and non-enzymatic antioxidant capacity (NEAC), cell lysates were prepared using RIPA buffer. The lysates were subsequently centrifuged at 12,000× *g* for 5 min to remove cellular debris, and the supernatants were collected for further analyses (Figure 8).

### 4.5. Measurement of Membrane Fluidity

Membrane fluidity was assessed using two fluorescent probes: 1,6-diphenyl-1,3,5-hexatriene (DPH) and 1-(4-trimethylammoniumphenyl)-6-phenyl-1,3,5-hexatriene p-toluenesulfonate (TMA-DPH). Following 24 h of exposure to ACR or GO the cells were washed twice with PBS then suspended in PBS (2 × 10^4^ cells/mL). DPH and TMA-DPH were added from 10 mM stock solutions (3 µL per 3 mL of cell suspension), yielding a final probe concentration of approximately 10 µM. Cells were incubated with the respective probe for 45 min, and fluorescence anisotropy measurements were performed at 37 °C. TMA-DPH and DPH probes localize in different regions of the plasma membrane, allowing the assessment of changes in membrane organization at distinct membrane depths. DPH primarily reflects the hydrophobic core of the lipid bilayer, whereas TMA-DPH is located closer to the membrane surface. Fluorescence anisotropy measurements were performed at 37 °C using a Varian Cary Eclipse spectrofluorometer. The G-factor was experimentally determined on the Cary Eclipse spectrofluorometer under the same optical conditions using untreated control samples as $G=\frac{I_{\mathrm{HV}}}{I_{\mathrm{HH}}}$ (where I_HV_ and I_HH_ represent the fluorescence intensities recorded with horizontal excitation and vertical or horizontal emission polarization, respectively), yielding values of approximately 1.02–1.06. For both DPH and TMA-DPH, fluorescence anisotropy values were determined using the standard equation at an excitation wavelength of 360 nm and an emission wavelength of 430 nm [34,35].

$$r=\frac{I∥-\mathrm{GI}⊥}{I∥+2\mathrm{GI}⊥}$$

where I‖—the intensity of the probe fluorescence is measured vertically, I⊥—the intensity of the probe fluorescence is measured horizontally, and G—correction factor.

### 4.6. Determination of ROS (Reactive Oxygen Species) and RNS (Reactive Nitrogen Species) Levels

Intracellular ROS/RNS-associated fluorescence was assessed using H_2_DCF-DA, DHEt, and DAF-FM after 24 h of exposure to ACR or GO. After incubation the cells were washed twice with PBS then suspended in PBS and transferred to 96-well plates containing modified Hanks’ balanced salt solution (HBSS). Thus, probe loading was performed under serum-free conditions. H_2_DCF-DA, DHEt, and DAF-FM were each used at a final concentration of 5 µM, and cells were incubated with the respective probe for 30 min at 37 °C. No probenecid or other efflux inhibitor was used during probe loading or fluorescence measurements. Fluorescence intensity was measured using a Varian Cary Eclipse fluorescence microplate reader at the following excitation/emission wavelengths: H_2_DCF-DA-derived fluorescence, λ_ex_ = 504 nm and λ_em_ = 529 nm; DHEt, λ_ex_ = 528 nm and λ_em_ = 605 nm; and DAF-FM, λ_ex_ = 495 nm and λ_em_ = 515 nm. The results are presented as relative fluorescence intensity and expressed as a percentage of the untreated control, which was set at 100% [36].

### 4.7. Determination of Free Thiol Group Content

Thiol group content was quantified using a fluorometric assay based on *o*-phthalaldehyde (OPA). PBMC lysates were combined with a redox-quenching buffer containing trichloroacetic acid, *N*-ethylmaleimide (NEM) in RQB, and potassium phosphate (KP) buffer (pH 7.0), followed by incubation at room temperature for 5 min. Subsequently, KP buffer (pH 6.9) and OPA were added, and the samples were incubated in the dark at room temperature for 30 min. Fluorescence generated by the OPA–thiol reaction was measured at an excitation wavelength of 365 nm and an emission wavelength of 430 nm. Thiol group levels were calculated from a calibration curve prepared using known concentrations of reduced glutathione as a standard and were expressed as nmol per mg of protein [37].

### 4.8. Determination of Glutathione (GSH) Content

The reduced GSH concentration was also determined using the fluorometric method with OPA. GSH levels were calculated from a calibration curve prepared using known concentrations of reduced glutathione as a standard and were expressed as nmol per mg of protein [36].

### 4.9. Determination of Free Amino Group Content

For determination of free amino groups in lysate proteins, 2,4,6-trinitrobenzenesulfonic acid (TNBS) was used. The reaction of TNBS with amines generates a colored product that can be readily measured at 335 nm. The content of amino groups was calculated based on the calibration curve for different concentrations of homocysteine and expressed as nmol/mg protein [38].

### 4.10. Determination of Total Non-Enzymatic Antioxidant Capacity (NEAC)

NEAC of PBMCs was determined using the FRAP (Ferric Reducing Antioxidant Power) method described by Benzie and Strain [39]. Reduction of the Fe(III)–TPTZ complex to Fe(II)–TPTZ was measured spectrophotometrically at 593 nm, and the results were expressed as nmol Trolox equivalents per mg protein.

### 4.11. Measurement of Glutathione Peroxidase (GPx) Activity

GPx activity in PBMCs was determined spectrophotometrically following the Sigma-Aldrich glutathione peroxidase assay kit (Catalog Number: MAK437). The GPx activity in the cells was expressed as U/mg protein.

### 4.12. Measurement of Catalase (CAT) Activity

CAT activity in PBMCs was determined spectrophotometrically following the method of Aebi using hydrogen peroxide as a substrate [40]. The decomposition of hydrogen peroxide was measured at 240 nm and 25 °C for 1 min. The CAT activity in the cells was expressed as U/mg protein.

### 4.13. Superoxide Dismutase (SOD1) and Catalase (CAT) Expression

To evaluate the expression levels of SOD1, CAT, GAPDH, PBMCs exposed to ACR and GO were lysed in RIPA buffer. Following centrifugation, the resulting supernatants were collected, and equal amounts of lysate protein (25 µg) were separated by SDS-PAGE and transferred onto Immobilon-P membranes according to the method described by Towbin et al. After blocking with 5% non-fat dry milk in TBST for 1 h, the membranes were incubated overnight at 4 °C with primary antibodies specific for SOD1, CAT, GAPDH diluted in TBST. Alkaline phosphatase-conjugated secondary antibodies were applied for 2 h at room temperature, and the bands were visualized using the appropriate substrate [41,42]. Chemiluminescent signals were detected and captured using an Azure 300 Imaging System (Azure Biosystems, Dublin, CA, USA). Full botts in Supplementary Materials.

### 4.14. Determination of Protein Concentration

The protein concentration in PBMCs lysates was determined using the method of Lowry et al. [43]. The method measures protein concentration against an albumin standard curve by reducing Cu^2+^ in alkaline conditions, followed by Folin–Ciocalteu reagent reduction to molybdenum blue (absorbance peak at 750 nm).

### 4.15. Statistical Analysis

Statistical analysis of the data involves checking the normality of the distribution of the parameters tested using the Shapiro–Wilk test. Then, the homogeneity of variances was checked using Levene’s test. A one-way ANOVA with Tukey’s post hoc test for multiple comparisons was used for data that followed a normal distribution and exhibited homogeneity of variance. In other cases, the nonparametric Kruskal–Wallis test was used to compare differences between the tested groups. Statistical significance was accepted at *p* < 0.05. Each measurement was conducted 3 to 8 times in independent experiments, with 3 to 6 replicates in each. Statistical analysis was performed using Statistica v.13.3 (StatSoft Polska, Krakow, Poland).

## 5. Conclusions

The concentrations of ACR and GO used in this study were much higher than those typically observed under physiological conditions. The results should be interpreted mainly as a mechanistic model of increased carbonyl stress in PBMCs. Despite this limitation, both aldehydes clearly disrupted redox homeostasis by increasing ROS/RNS production, depleting GSH, reducing non-enzymatic antioxidant capacity, and decreasing free thiol and amino group levels in cellular proteins. The observed reduction in CAT and GPx activity may result from altered protein expression, oxidative modification, or direct interactions of ACR and GO with enzyme functional groups. Overall, although ACR and GO differ in chemical structure and toxic potency, both induced convergent effects involving carbonyl stress, antioxidant depletion and oxidative/nitrosative imbalance. ACR showed stronger cytotoxicity at micromolar concentrations, whereas GO required millimolar concentrations to produce comparable effects. Further studies using lower, chronic exposure models are needed to better reflect physiologically relevant conditions.

## Data Availability

The original contributions presented in this study are included in the article/Supplementary Material. Further inquiries can be directed to the corresponding author.

## Supplementary Materials

The following supporting information can be downloaded at: https://www.mdpi.com/article/10.3390/ijms27198706/s1.

## Abbreviations

The following abbreviations are used in this manuscript:

ACR	acrolein
AGEs	advanced glycation end-products
ARE	antioxidant response element
CAT	catalase
DAF-FM	4-amino-5-methylamino-2′,7′-difluorofluorescein
DHEt	dihydroethidium
DNPH	2,4-dinitrophenylhydrazine
DPH	1,6-diphenyl-1,3,5-hexatriene
DTNB	5,5′-dithiobis(2-nitrobenzoic acid)
FBS	fetal bovine serum
FRAP	ferric reducing antioxidant power
GAPDH	glyceraldehyde-3-phosphate dehydrogenase
GO	glyoxal
GPx	glutathione peroxidase
GSH	reduced glutathione
H_2_DCF-DA	2′,7′-dichlorodihydrofluorescein diacetate
LDL	low-density lipoprotein
NEAC	non-enzymatic antioxidant capacity
NEM	N-ethylmaleimide
Nrf2	nuclear factor erythroid 2-related factor 2
OPA	o-phthalaldehyde
PBMCs	peripheral blood mononuclear cells
PBS	phosphate-buffered saline
PMS	phenazine methosulfate
PVDF	polyvinylidene fluoride
RAGE	receptor for advanced glycation end-products
RCS	reactive carbonyl species
RIPA	radioimmunoprecipitation assay buffer
RNS	reactive nitrogen species
ROS	reactive oxygen species
RPMI 1640	Roswell Park Memorial Institute 1640 medium
SDS-PAGE	sodium dodecyl sulfate–polyacrylamide gel electrophoresis
SOD1	superoxide dismutase
TBST	Tris-buffered saline containing Tween 20
TMA-DPH	1-(4-trimethylammoniumphenyl)-6-phenyl-1,3,5-hexatriene p-toluenesulfonate
TNBS	2,4,6-trinitrobenzenesulfonic acid
TPTZ	2,4,6-tripyridyl-s-triazine
XTT	2,3-bis(2-methoxy-4-nitro-5-sulfophenyl)-2H-tetrazolium-5-carboxanilide
