# Supplementary material for: Comparative Effects of Acrolein and Glyoxal on Redox Homeostasis, Antioxidant Defense and Membrane Properties in Human Peripheral Blood Mononuclear Cells: An In Vitro Study
Source: Int J Mol Sci. 2026 Sep 29;27(19):8706. doi: 10.3390/ijms27198706 (PMC13665209; doi:10.3390/ijms27198706)

CATALASE

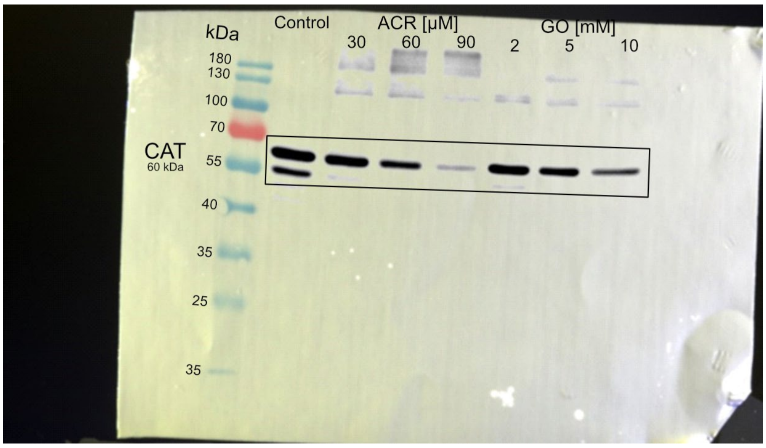

CATALASE GAPDH

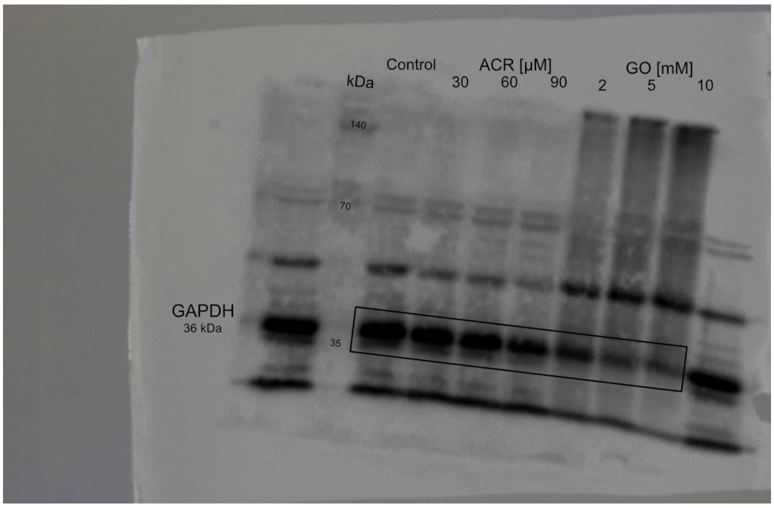

SOD1

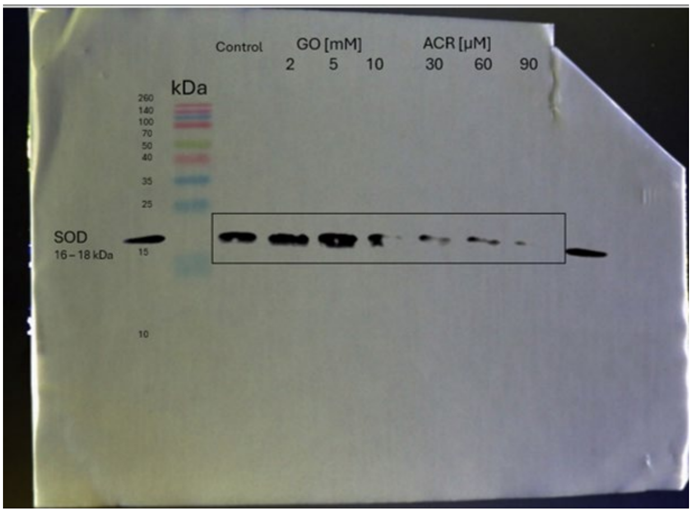

SOD1 GAPDH

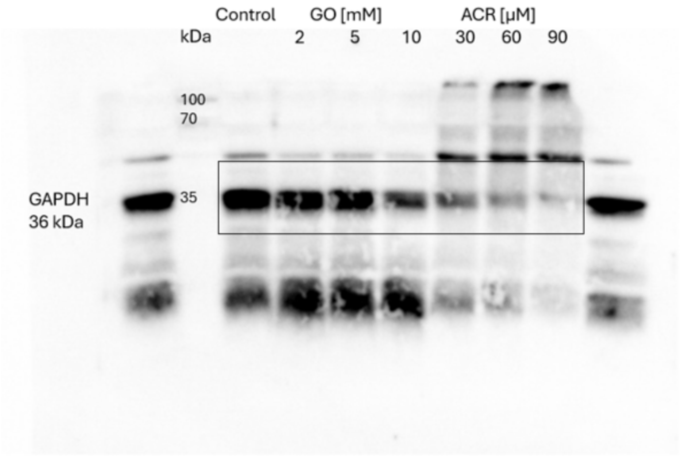

PONCEAU S

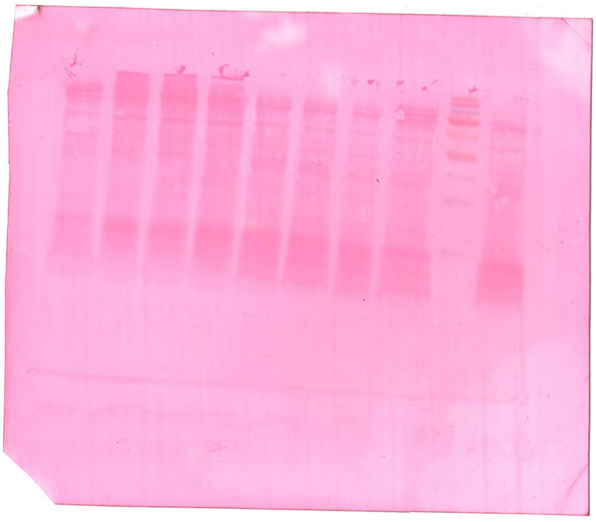

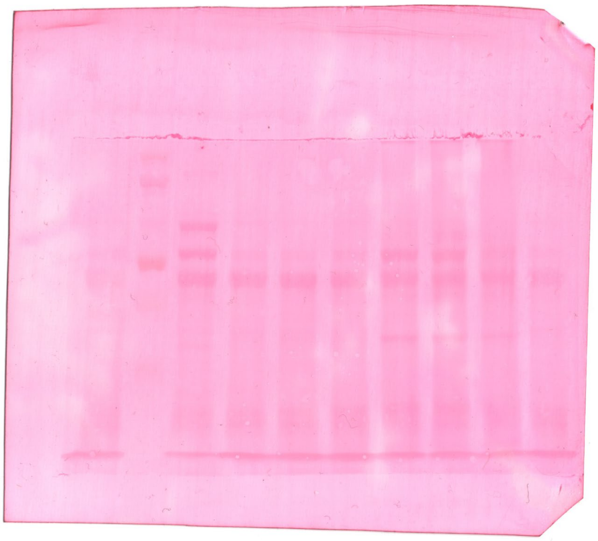

Supplement: Supplementary file 1 [file ijms-27-08706-s001.zip › ijms-4572217-supplementary.pdf]
